# Serum and Seminal Plasma Zinc Levels and Immunopositivity of the ZIP6 and ZIP14 Transporters in Men with Normo- and Teratozoospermia

**DOI:** 10.3390/cimb47020101

**Published:** 2025-02-06

**Authors:** Aleksandra Veselinović, Aleksandar Stojsavljević, Aleksandra Arsić, Dragana Bojović-Jović, Vesna Vučić, Igor Golić

**Affiliations:** 1 Cognitive Neuroscience Department, Research and Developmental Institute “Life Activities Advancement Institute”, 11000 Belgrade, Serbia; a.veselinovic@add-for-life.com; 2 Department of Speech, Language and Hearing Sciences, Institute for Experimental Phonetics and Speech Pathology, 11000 Belgrade, Serbia; 3 Innovative Centre, Faculty of Chemistry, University of Belgrade, 11000 Belgrade, Serbia; aleksandars@chem.bg.ac.rs; 4 Group for Nutritional Biochemistry and Dietology, Institute for Medical Research, National Institute of Republic of Serbia, University of Belgrade, 11000 Belgrade, Serbia; aleksandraarsicimi@gmail.com (A.A.); vesna.vucic.imr@gmail.com (V.V.); 5 Center of Research Excellence in Nutrition and Metabolism, Institute for Medical Research, National Institute of Republic of Serbia, University of Belgrade, 11000 Belgrade, Serbia; 6 ART Department, Clinic for Gynaecology and Obstetrics “Narodni Front”, 11000 Belgrade, Serbia; drbojovicjovic@gmail.com; 7 Center for Electron Microscopy, Faculty of Biology, University of Belgrade, 11000 Belgrade, Serbia

**Keywords:** zinc, ZIP transporters, male infertility, normozoospermia, teratozoospermia

## Abstract

Zinc plays a crucial role in spermatogenesis, sperm function, and fertilisation. Zinc homeostasis is regulated by ZIP and ZnT transporter proteins, which mediate Zn^2+^ influx and efflux across sperm cell membranes. This study analysed total Zn concentration in seminal plasma and serum of 10 normozoospermic and 32 teratozoospermic men involved in the process of infertility treatment, using inductively coupled plasma mass spectrometry. In addition, the expression of Zn transporters ZIP6 and ZIP14 in the sperm of two normozoospermic and two teratozoospermic men was analysed using immunofluorescence. Applying Student’s *t* test and the Mann–Whitney U test, we found no significant differences in Zn concentrations in seminal plasma and serum between groups. ZIP6 was mainly localised in the sperm head, with slightly higher immunopositivity in normozoospermic than teratozoospermic samples, but there was no statistically significant difference between the groups. ZIP14 was mainly found in the sperm head, and some teratozoospermic samples showed immunopositivity in the tail, although there were no significant differences in ZIP14 immunopositivity between normozoospermic and teratozoospermic samples. The results suggest that Zn concentrations in seminal plasma and serum, and the expression of ZIP6 and ZIP14, do not differ in normo- and teratozospermic samples, and emphasise the complex interplay of factors underlying male fertility.

## 1. Introduction

Infertility remains a serious global health problem, affecting an estimated 8–12% of couples of reproductive age. Male factors alone are responsible for about 30% of infertility cases, with a further 20% being a contributory cause [1]. Treatments are available for many types of male infertility; however, when conventional treatments are not indicated or prove ineffective, assisted reproductive technology (ART) can be recommended [2]. Epidemiological studies have shown that sperm quality has declined markedly in recent decades. That emphasises the importance of comprehensive investigations—including medical and reproductive history, physical examination, semen analysis, and testicular biopsy (if necessary) [3]. The World Health Organization (WHO) [4] recommends conventional semen analysis as an initial step in the assessment of male (in)fertility, although its accuracy in determining fertility potential or predicting reproductive success is limited.

Trace elements play a crucial role in the male reproduction process [5]. One of the essential trace elements for male fertility is zinc (Zn), which has various roles in the male reproductive system, including regulating the process of spermatogenesis, epididymal sperm maturation, sperm structure and function, sperm motility, capacitation, fertilisation, and embryonic development [6,7].

To fulfil its diverse bioactive functions, Zn^2+^ requires specialised systems to facilitate its transport across biological membranes [6]. Zinc ions, due to their hydrophilic nature and positive charge, cannot readily cross the lipid bilayer of the plasma membrane or intracellular membranes [8]. The cellular import and export of Zn^2+^ are facilitated by two families of Zn^2+^ transport proteins conserved in mammals: SLC39s (ZIPs), which enable the influx of Zn^2+^ into the cytoplasm; and SLC30s (ZnTs), which mediate the efflux of Zn^2+^ from the cytosol [9]. These transporters also facilitate moving the Zn^2+^ in opposite directions across cellular and intracellular membranes, playing vital roles in a various physiological and cellular processes—including immune, endocrine, reproductive, skeletal, and neuronal functions—all achieved through the precise regulation of Zn^2+^ homeostasis [9].

During the mitotic phase of spermatogonia and the meiotic phase of spermatocytes, Zn^2+^ plays a crucial role in facilitating ribonuclease activities. As spermatogenesis progresses, Zn^2+^ accumulates in germ cells and increases in the testes, highlighting its importance in the development of sperm [10]. Throughout this process, spermatids undergo significant morphological transformations, developing a tail for motility and a mid-piece that connects the head to the tail. After the spermatids complete their differentiation, mature sperm are released from the Sertoli cells into the lumen of the seminiferous tubules [11]. The survival of germ cells and the proper replacement of protamines during spermatogenesis are heavily dependent on Zn^2+^ [12], particularly during the final stages of spermatid differentiation, where it assists in packaging DNA within the nucleus. It binds to protamines to form disulphide bonds that stabilise the chromatin structure, ensuring proper DNA condensation and organisation during sperm development. Eventually, Zn^2+^ becomes highly concentrated in the tail region of mature spermatozoa, significantly contributing to sperm motility [10]. ZnT and ZIP transporters are essential for maintaining the supply and balance of Zn^2+^. ZIP proteins are located on the plasma membrane of Sertoli cells, where they acquire Zn^2+^ from the circulation. Subsequently, ZnT is responsible for exporting Zn^2+^ to developing germ cells [12,13].

In the study of Croxford et al. (2011) conducted on mice, Zn^2+^ importer localisation was investigated in specific cell types, where ZIP6 was localised to round and elongating spermatids, required for different stage-specific processes (such as facilitating Zn^2+^ import from prostatic fluid for viability and/or motility once spermatozoa are ejaculated), while ZIP14 was present in spermatogonia [13]. Foresta et al. (2014) investigated the dynamics of Zn transport in human sperm cells throughout their development and maturation and showed that ZIP6 transporters are present at all stages of spermatogenesis, while ZIP14 was not found in this study [14]. However, in a more recent study by Protić et al. (2022), the presence of ZIP14 was found in human normozoospermic and asthenozoospermic samples [15].

The aim of this study was to compare Zn levels in serum and seminal plasma of 42 normo- and teratozoospermic men divided into the two groups: normozoospermic men (N) (n = 10) and teratozoospermic men (T) (n = 32). Also, in order to characterise immunoexpression of ZIP6 and ZIP14 transporters in ejaculated spermatozoa, we selected four representative samples (two normozoospermic and two teratozoospermic) with achieved pregnancy as a pilot study. The representative samples were randomly selected to minimise bias and ensure that they accurately reflected the characteristics of each study group.

## 2. Materials and Methods

### 2.1. Collection and Preparation of Samples

This cross-sectional study was conducted at the Department of Assisted Reproductive Techniques of the Clinic of Gynaecology and Obstetrics “Narodni Front” in Belgrade, Serbia. The study received approval from the Ethics Committee of the Clinic for Gynaecology and Obstetrics “Narodni Front” in Belgrade, Serbia (No. 05006-2022-19144, 4.11.2022), ensuring that all procedures adhered to ethical standards and that participant data privacy was protected.

The participants were selected based on the following inclusion criteria: absence of azoospermia, sperm leukocytes < 1 × 10^6^/mL, sexual abstinence for 3–5 days before semen collection, and absence of genital infections (e.g., urethritis, prostatitis, sexually transmitted diseases) and systemic diseases (e.g., diabetes, cancer, autoimmune diseases). After the men met these criteria and signed a consent form to participate in the study, they donated semen samples in accordance with the WHO recommendations 2021 [4]. The semen samples were delivered to the clinic in sterile containers and analysed in the same laboratory. After liquefaction at 37 °C for 20 min, a routine analysis of the semen (liquefaction time, volume, pH, viscosity, sperm count, motility, and morphology) was carried out, according to the WHO guidelines from 2021. The criteria for sperm normality (normozoospermia) were as follows: sperm concentration ≥ 16 × 10^6^ million/mL of ejaculate; total sperm number > 39 million per ejaculate; progressively motile sperms ≥ 30%, and normal sperm morphology ≥ 4%. Samples with sperm concentration ≥ 16 × 10^6^/mL of ejaculate, total sperm number > 39 million per ejaculate, motility ≥ 30% and morphology < 4% were considered teratozoospermic. The samples with combined semen abnormalities (e.g., oligoteratozoospermia and astenoteratozoospermia) were excluded from analysis.

### 2.2. Quantification of Zn

A singular standard solution of Zn at a concentration of 10 mg/L (Merck, Darmstadt, Germany) was used to generate six intermediate standard solutions. The resulting calibration curve exhibited a linearity exceeding 0.999. To address matrix interferences, a Ge internal standard solution at a concentration of 10 mg/L was utilised following a final dilution to 10 μg/L. This solution was uniformly dispensed across blanks, standard solutions, and samples via a secondary channel of the peristaltic pump. Zinc quantification was conducted using inductively coupled plasma mass spectrometry (ICP-MS, iCAP Q_c_, Thermo Scientific, London, UK). The accuracy of the analytical method was verified using the standard reference material (SERO210105, Level-1, supplied by Seronorm, Sero AS, Hvalstad, Norway); measured values of the selected ^66^Zn isotope agreed between 94.5 and 102.2% with the declared values of the standard reference material.

### 2.3. Immunofluorescence

For immunofluorescence detection, native sperm smears on SuperFrost Plus slides were first fixed in methanol for 15 min and then air-dried. The smears were then fixed and permeabilised in acetone for 5 min and additionally permeabilised with 0.3% Triton X-100 in Tris-buffered saline (TBS) for 10 min. To block non-specific binding, slides were incubated for 30 min with a blocking solution of 0.1% bovine serum albumin, 3% normal goat serum, 0.2% Triton X-100 and 0.05% Tween-20 in TBS. The smears were then incubated overnight at 4 °C with the primary antibodies against ZIP6 (1:50, PA5-21071, Invitrogen, Carlsbad, CA, USA) and ZIP14 (1:50, PA5-21077, Invitrogen, Carlsbad, CA, USA) in the same blocking buffer. The next day, the slides were washed three times in TBS with 0.05% Tween-20 (TBS-T) before labelling with the secondary antibody Alexa Fluor 488 (1:400, A-11029, Invitrogen, Carlsbad, CA, USA) for one hour at room temperature. After labelling, slides were rinsed twice in TBS-T and once in TBS, and then counterstained with Sytox Orange nuclear stain (1:1000, S11368, Life Technologies, Gaithersburg, MD, USA) for 5 min. Finally, the slides were rinsed in TBS and mounted with Mowiol (Sigma-Aldrich, Steinheim, Germany) [16]. All chemicals, unless otherwise stated, were purchased from Sigma-Aldrich (Steinheim, Germany).

Images were acquired with a Leica TCS SP5 II confocal microscope (Leica Microsystems, Wetzlar, Germany) in sequential mode to prevent channel crosstalk. Smears were excited with a 488 nm laser, and nuclei were visualised with a 543 nm laser, using blue false colouring applied to clearly distinguish the green channel. The specificity of the immunofluorescence was validated by omitting the primary antibodies. In parallel, negative controls were performed to detect non-specific staining.

The number of immunopositive spermatozoa was quantified using the multipoint tool of ImageJ software version 1.54m (NIH, Bethesda, MD, USA) and expressed as a percentage. For each sample type, six randomly selected fields were analysed, with each field containing over 50 spermatozoa.

### 2.4. Statistics

Statistical analysis was performed using SPSS statistical package (v. 22.0; IBM, Chicago, IL, USA) and Prism 8, version 8.4.3. (GraphPad Software, San Diego, CA, USA). To assess normality distribution data, the Shapiro–Wilk test was used. Data are shown as mean ± standard deviation (SD) for continuous variables with normal distribution or as median and interquartile range for non-normally distributed data. For comparisons between groups, Student’s *t* test and the Mann–Whitney U test were applied, depending on the normality of the variables. Additionally, the Student’s *t* test was used to test the overall immunopositivity of the sperm samples and the significance of differences between normozoospermic and teratozoospermic samples. Statistical significance was considered at *p* < 0.05.

## 3. Results

Demographic and seminogram parameters between groups are presented in Table 1. The results show no significant differences in age, body mass index (BMI), sperm volume, sperm concentration, or total sperm number between the normozoospermic (N) and teratozoospermic (T) groups. There were significant differences in sperm morphology and sperm progressive motility between the N and T groups.

Table 2 presents the Zn levels in serum and seminal plasma, showing no significant differences between the N and T groups, although slight trends can be observed.

No statistically significant differences in Zn concentration in serum and seminal plasma were found between the groups.

Immunofluorescent detection of ZIP6 and ZIP14 was conducted on two normozoospermic and two teratozoospermic samples with achieved pregnancy. Sperm cells revealed the presence of ZIP6 and ZIP14 expression mostly within the head of the spermatozoa.

### 3.1. ZIP6

**Figure 1:**
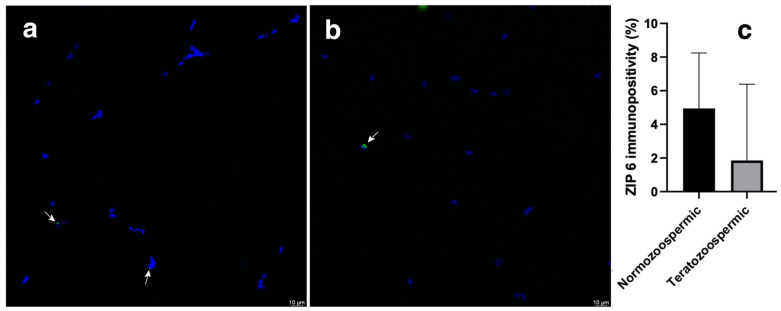
Immunofluorescence detection of ZIP6 transporter in normozoospermic (**a**) and teratozoospermic (**b**) sperm samples using a confocal laser scanning microscope. ZIP6 transporter is shown in green, white arrows indicate its position, and sperm nuclei are counterstained in blue. Scale bar: 10 μm. Relative ZIP 6 immunopositivity among the heads of normozoospermic and teratozoospermic sperm samples (**c**). For each type of sample, six randomly selected field areas were analysed (>50 cells). The results are the means ± SD.

In normozoospermic samples, the ZIP6 was mostly found in the sperm head (Figure 1a). In teratozoospermic samples, where abnormal spermatozoid morphology was observed as expected, the ZIP6 immunopositivity was predominantly noticed in the sperm head (Figure 1b). No presence of ZIP6 in the tail of normozoospermic and teratozoospermic samples was observed.

Normozoospermic samples had a mean value 4.94 ± 3.29% ZIP6 immunopositivity in the head of spermatozoa, while teratozoospermic samples had a mean 1.85 ± 4.53% ZIP6 immunopositivity in the head of spermatozoa (Figure 1c).

Although the numerical difference in the means was present, the results of Student’s *t* test on the ZIP6 immunopositivity between normozoospermic and teratozoospermic showed no statistically significant difference.

### 3.2. ZIP14

**Figure 2:**
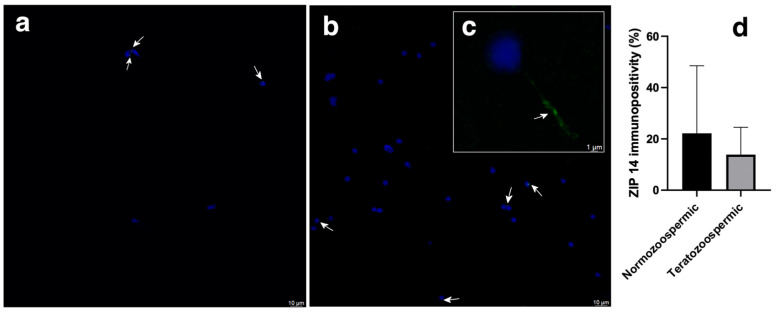
Immunofluorescence detection of ZIP14 transporter in the normozoospermic (**a**) and teratozoospermic (**b**) sperm samples, and in the tail of teratozoospermic sperm samples using a confocal laser scanning microscope (inset, (**c**)). ZIP14 transporter is shown in green, white arrows indicate its position, and sperm nuclei are counterstained in blue. Scale bar: 10 μm. Relative ZIP 14 immunopositivity among the heads of normozoospermic and teratozoospermic sperm samples (**d**). For each type of sample, six randomly selected field areas were analysed (>50 cells). The results are the means ± SD.

The majority of the ZIP14 immunostaining was observed in the head of both normozoospermic and teratozoospermic samples (Figure 2a,b). However, ZIP14 immunostaining was observed in the tail of certain spermatozoa within teratozoospermic samples (Figure 2c).

Normozoospermic samples had a mean 22.1 ± 26.3% of ZIP14 immunopositivity in the head of spermatozoa, whereas teratozoospermic samples had a mean 13.8 ± 10.6% ZIP14 immunopositivity in the head of spermatozoa (Figure 2d). However, comparison of ZIP14 immunopositivity between normozoospermic and teratozoospermic men did not reveal a statistically significant difference.

## 4. Discussion

Male infertility is influenced by a number of factors, some of which are not yet fully understood, making it a complex global health problem. Zinc, an essential trace element for sperm activity, is required in certain amounts to enable acrosome reaction, motility, and capacitation [10]. Since both an excess and a deficiency of Zn can impair sperm function, controlling the level of Zn in sperm is crucial for the best chance of fertilisation [7].

Many studies show that higher levels of Zn in seminal plasma or serum are associated with normozoospermia and better semen quality, which correlate significantly with sperm count, motility, normal morphology, and fertility [17,18,19,20,21,22]. Subsequently, we examined total Zn concentration in seminal plasma and serum from normozoospermic and teratozoospermic samples.

The participants in our study had matched age and BMI. Some differences were found in seminogram parameters, as expected. However, no statistically significant difference was found in serum and seminal Zn levels between normozoospermic and teratozoospermic men. Nenkova et al. (2017) examined seminal plasma Zn levels in normozoospermia and teratozoospermia, and found mean values about 150 mg/L and 127 mg/L, respectively [17]. The median values found in our study were about 60,109 μg/L (60.1 mg/L) for normozoospermia and 63,399 μg/L (63.3 mg/L) for teratozoospermia, which is a half of the concentrations found by Nenkova et al. [17]. This could be due to the Zn deficiency that has been previously reported in the Serbian adult population [23,24]. Stojsavljević et al. (2020) investigated Zn levels in serum and blood samples in the adult population of the Republic of Serbia, and found the presence of Zn in serum in the range 339–871 μg/L [24], elucidating that Zn content was higher in men than women, with the noticed trend of increasing with age. This is in line with the results of our study, where mean Zn values in serum were about 670 μg/L in normozoospermic and 658 μg/L in teratozoospermic samples. Furthermore, Jagodić et al. (2019) found a serum Zn value in the general adult population of about 574 μg/L, and reported that the mean Zn serum value was lower compared to results from Poland (824 μg/L), Italy (732 μg/L), Germany (830 μg/L), Greece (741 μg/L) [25], France (928 μg/L), and Sweden (728 μg/L) [23]. Therefore, low levels of Zn in serum and seminal plasma in all participants, both with normozoospermia and teratozoospermia, and a lack of differences in these two groups, could be explained by living in a Zn-deficient area.

A modest number of studies have examined Zn concentrations in the bloodstream (serum/plasma) and/or seminal plasma and semen quality and/or fertility rate between normo- and teratozoospermic men. However, the results among studies are uneven. Atig et al. (2012) found significantly higher seminal Zn levels in 60 normozoospermic men (144 ± 42.1 mg/L) than in all three groups of infertile men, including a group of 60 teratozoospermic men [19]. Nenkova et al. (2017) reported that seminal Zn levels were significantly lower in groups of men with poor semen quality (teratozoospermia, asthenoteratozoospermia, and oligoteratozospermia) than in a normozoospermic group [17]. Most importantly, a systematic review and meta-analysis by Zhao et al. (2016) reported that seminal plasma Zn levels from infertile men were significantly lower than those from normal/fertile controls, although some papers included showed no differences in seminal Zn levels between fertile and infertile men [26]. It should be noted that in this study, we compared normozoospermic and teratozoospermic samples, and not fertile and infertile men, since the teratozoospermic patients achieved pregnancy.

Further, we examined immunolocalisation of the transporters ZIP6 and ZIP14 in ejaculated sperm from normozoospermic and teratozoospermic samples with achieved pregnancy outcomes as a pilot study. Our results show that ZIP6 is immunopositive in the head of normozoospermic and teratozoospermic spermatozoa, which is consistent with the study by Protić et al., who identified ZIP6 mainly in the head of normozoospermic and asthenozoospermic spermatozoa [15]. The absence of ZIP6 in the subcompartments mentioned by Protić et al. could be due to methodological differences, as native smear preparations, in contrast to processed gradient methods, may not sufficiently expose certain subcompartments that are crucial for the detection of ZIP6. The absence of ZIP6 in the tail raises the question of the role of Zn in sperm motility, which remains unclear. Future studies using functional assays are needed to confirm these roles and clarify the involvement of ZIP6 in sperm motility and fertility.

Given the typical morphological abnormalities in teratozoospermia, which include defects at the sperm head, the presence of ZIP6 in these defective spermatozoa suggests a possible link between ZIP6 expression and sperm maturation. The process of chromatin integrity in male germ cells is crucial for sperm functionality and male fertility [27]. Chromatin structure is packed to be extremely resistant to DNA damage in the sperm and rapidly decondensed to make the DNA available in the ooplasm after fertilisation [22,28]. Proper regulation of Zn is critical for nuclear stability, and intracellular Zn^2+^ dynamics activate Zn signalling by regulating the expression of stage-specific transporters [13]. Further studies may reveal whether ZIP6 expression serves as a maturation marker and whether it is related to nuclear Zn^2+^ concentration and chromatin stabilisation, both of which are critical for fertilisation [15,29].

Our study showed that ZIP14 is localised in the sperm head of normozoospermic and teratozoospermic samples and in the tail of teratozoospermic samples, indicating a context-dependent role of ZIP14 in sperm physiology. ZIP14 is present in the sperm head, suggesting a possible involvement in chromatin stabilisation and acrosome function, which is important for oocyte penetration. The localisation in the tail in teratozoospermic samples may be a response to morphological defects, possibly as a compensatory mechanism under conditions of suboptimal Zn^2+^ homeostasis. This role of ZIP14 in Zn^2+^ regulation, particularly under stress or abnormal conditions, is consistent with its function in systemic Zn^2+^ homeostasis during inflammation, in which ZIP14 is upregulated by inflammatory cytokines [30]. Further investigation of the ZIP14 functional versatility in response to sperm abnormalities could elucidate its potential compensatory role in maintaining Zn^2+^ balance.

Therefore, the localisation of ZIP6 and ZIP14 in sperm suggests certain functional implications in Zn homeostasis and sperm function. The presence of ZIP6 in the sperm head may suggest that it is involved in the regulation of nuclear Zn^2+^ concentration, which is essential for chromatin stabilisation and the maintenance of DNA integrity during fertilisation [15]. Its absence in the tail raises questions about the role of Zn^2+^ in motility, possibly pointing to other mechanisms regulating Zn^2+^ in this region. Similarly, the presence of ZIP14 in the sperm head fits with its possible role in facilitating fertilisation entry through chromatin stabilisation and acrosome function. In contrast, the presence of ZIP14 in the tail in teratozoospermic samples could represent a compensation process to remedy the disturbed Zn^2+^ homeostasis associated with morphological defects.

The absence of ZIP transporters in the sperm midpiece—a region central to Zn^2+^ storage and mitochondrial activity—raises interesting questions about alternative Zn^2+^ regulatory mechanisms in this compartment. The midpiece plays a central role in energy metabolism by modulating ATP synthesis and oxidative phosphorylation, which are important for motility [6,31]. Despite its apparent importance, there were no Zn^2+^ transporters in this region, suggesting that mitochondrial Zn^2+^ homeostasis may be regulated by pathways other than ZIP or that Zn^2+^ is controlled by transient release from other compartments. Future studies should aim to confirm Zn^2+^ dynamics in the midpiece using advanced imaging techniques and biochemical assays, as excessive Zn^2+^ accumulation may lead to oxidative stress and impaired mitochondrial function [32]. Furthermore, variations in the immunoexpression of Zn^2+^ transporters are not necessarily associated with a specific semen abnormality, but with individual variations. Zinc deficiency can have various causes, including genetic background, and since the Zn^2+^ transporters have been identified as specialised transmembrane proteins that maintain cellular zinc homeostasis [33], their role in individual Zn metabolism should be further investigated.

The study’s limitations include the small sample size due to sample availability and ethical considerations, and the use of native smear preparations, which could potentially impact the accuracy and generalisability of the transporter localisation results. Furthermore, although native smears may help preserve cells in a more natural state, they might not entirely replicate in vivo conditions, which could introduce artifacts in localisation patterns. A limited sample size may reduce statistical power and increase variability, challenging the detection of subtle trends or drawing of definitive conclusions. These limitations suggest that while the results provide valuable insights, further studies with larger sample sizes, including diverse cohorts encompassing a wider range of age groups and clinical characteristics, would help to confirm and extend these results. In addition, the use of alternative methodologies, such as single-cell RNA sequencing to reveal cellular heterogenity and advanced imaging techniques, would allow a more comprehensive understanding and validation of the findings. Nevertheless, this research is among the first to examine ZIP transporters in relation to normo- and teratozoospermic types of seminograms in a human model.

## 5. Conclusions

This study has shown that serum and seminal plasma Zn levels did not differ significantly between normozoospermic and teratozoospermic men, and that both groups had Zn levels significantly below the levels in other countries. The general Zn deficiency can explain the lack of differences in serum and seminal plasma Zn values between the two study groups. Further, the immunoexpression of ZIP6 and ZIP14 in ejaculated spermatozoa was also similar in normo- and teratozoospermic samples that have achieved pregnancy, hinting that the results of our study are limited to men with (their female partners’) achieved pregnancy outcomes and do not exclude the possibility that zinc transporters may affect fertilisation in other populations. Future studies with larger cohorts involving broader populations are needed to more fully evaluate the role of zinc transporters in reproductive outcomes.

## Figures and Tables

**Table 1:** Demographic and seminogram characteristics of participants in the groups.

Seminogram Parameters	N (n = 10) Mean ± SD	T (n = 32) Mean ± SD	*p*
**Age, years**	36.6 ± 2.60	37.8 ± 5.41	*p* = 0.729
**BMI, kg/m^2^**	27.3 ± 1.62	27.1 ± 3.43	*p* = 0.838
**Sperm volume**	2.76 ± 0.96	2.85 ± 1.37	*p* = 0.416
**Sperm concentration (** **×** **10^6^/mL)**	30.8 ± 11.9	43.9 ± 16.6	*p* = 0.176
**Total sperm number (** **×10^6^** **)**	85.8 ± 45.6	112.9 ± 57.1	*p* = 0.861
**Sperm progressive motility (%)**	38.5 ± 3.4	36.1 ± 3.1	*p* = 0.049 *
**Sperm normal morphology (%)**	41.4 ± 7.09	1.71 ± 0.76	*p* = 0.000 ***

N, normozoospermic group; T, teratozoospermic group; BMI, body mass index. Values are reported as mean ± SD; SD, standard deviation. Variables are presented as mean ± SD and compared by Student’s *t* test. * *p* < 0.05, *** *p* < 0.001.

**Table 2:** Zinc levels in serum and seminal plasma in the N and T groups.

	N Group (n = 10), Mean ± SD or Median (IQR)	T Group (n = 32), Mean ± SD or Median (IQR)	*p*
**Serum Zn level (** **μg/L** **)**	670.2 ± 137.2	658.0 ± 105.1	*p* = 0.510
**Seminal plasma Zn level (** **μg/L** **)**	60,109 (65,480)	63,399 (59,444)	*p* = 0.756

N, normozoospermic group; T, teratozoospermic group. Values are presented as mean ± SD and median (IQR); SD, standard deviation, IQR, interquartile range. Normally distributed continuous variables are presented as mean ± SD; variables with skewed distribution are presented as median and interquartile range. Variables were compared by Student’s *t* test or by Mann–Whitney U test, depending on normality distribution.

## Data Availability

Data are contained within the article.
